# Patterns of Care and Utilization of Radiation for Women With Good-Risk Ductal Carcinoma In Situ: A National Cancer Database Analysis

**DOI:** 10.7759/cureus.28223

**Published:** 2022-08-21

**Authors:** Benjamin Silver, Sarah Mattessich, Irini Yacoub, Brian Rhee, David Schreiber

**Affiliations:** 1 Radiation Oncology, State University of New York Downstate Medical Center, Brooklyn, USA; 2 Radiation Oncology, Summit Medical Group, Berkeley Heights, USA

**Keywords:** national cancer database and seer analyses, practice patterns, radiation fractionation, breast cancer management, ductal carcinoma in-situ

## Abstract

Purpose/objective(s)

Lumpectomy followed by whole-breast radiation therapy (WBRT) provides a 50% recurrence rate reduction in ductal carcinoma in situ (DCIS) patients when compared to lumpectomy alone. Certain factors increase the risk of recurrence, including higher nuclear grade, large size, age less than 50, and close margins. RTOG 9804 demonstrated a reduction in local failure after WBRT with the use of adjuvant radiation in women with "good-risk disease" (mammographically detected, measuring less than or equal to 2.5 cm, with a predominant nuclear grade of 1 or 2, and a margin of greater than or equal to 1 cm, or a negative re-excision). The purpose of this study is to retrospectively identify the patterns of care in women with low-risk DCIS utilizing the National Cancer Database (NCDB). We hypothesize that with the utilization of hypofractionation, there may be an increase in the delivery of RT for these "good-risk" patients.

Materials/methods

The National Cancer Database was queried to identify women treated with lumpectomy for <2.5 cm, nuclear grade 1 or 2 DCIS of the breast from 2004 to 2016. Data were collected regarding age, tumor size, endocrine therapy use, ER receptor status, race, insurance type, and distance from the treatment center. The distance was stratified into quartiles consisting of 0-3.9, 4-8, 8.1-15.8, and > 15.8 miles, respectively. Radiation fractionation was collected and categorized as hypofractionation, standard fractionation, or other if fractionation could not be ascertained. Clinical and patient-related factors were compared between patients who received radiation and those who received no radiation. The frequency distributions between categorical variables were compared using the Chi-square test. Multivariable logistic regression was used to identify covariables that impacted the receipt of radiation.

Results

The eligibility criteria were met by a total of 12,846 patients. Of those, 6,600 (51.4%) received adjuvant WBRT. On multivariable regression, patients whose tumors were ER (OR 1.24, P<0.001) and those who had not received endocrine therapy (OR 2.24, P<0.001) were more likely to receive WBRT. Factors less likely to receive WBRT included increasing age over 50 (age 50-65 OR 0.83, P<0.001; age>65 OR 0.58, P<0.001), and distance of >15.8 miles (OR 0.78, P<0.001). The fractionation technique was categorized as standard or hypofractionated in 52.2% of patients. Of those, the use of hypofractionation increased from 0.4% in 2004 to 8.9% in 2010 and to 53.8% in 2016.

Conclusion

This NCDB analysis demonstrated that patients who meet the RTOG 9804 criteria for "good-risk" DCIS are less likely to receive RT as time progresses despite an increase in the utilization of hypofractionation techniques. Overall, slightly more than half of these patients receive adjuvant RT.

## Introduction

Ductal carcinoma in situ (DCIS) is a well-known precursor to invasive breast cancer. If left untreated, the 30-year risk of invasive transformation is reported to be as high as 25-30% [1]. Therefore, patients are offered standard-of-care treatment with either breast conservation therapy (lumpectomy followed by whole breast radiation) or mastectomy alone [2].

While no head-to-head study exists for DCIS, findings from sentinel clinical trials of invasive breast cancer showed no significant difference in overall survival between the two treatment regimens, and both are routinely accepted to apply to the treatment of DCIS. There are, however, many DCIS-specific trials that investigate the role of radiation as part of breast conservation therapy. They demonstrated that the addition of radiation provides a 50% recurrence rate reduction when compared to lumpectomy alone and that about 50% of local failures in DCIS patients are invasive [3-8].

Investigators further aimed to identify prognostic factors for local failure. In general, factors associated with a lower risk of recurrence of either DCIS or invasive disease include lower nuclear grade, smaller tumor size, age > 50, and sufficiently negative surgical margins. RTOG 9804 defined "good-risk DCIS" as mammographically detected disease, tumor size <2.5 cm, predominant nuclear grade of 1 or 2, surgical margin >3 cm, or a negative margin on re-excision. Recently published, a 15-year follow-up demonstrated that conventional whole breast radiation provided a local control benefit in this group, with a reduction in local failure from 15.1% to 7.1% [9,10].

While there is an evident relative benefit of radiation in low-risk disease, the absolute local failure rate after lumpectomy alone is still reasonably low, leading physicians and patients to consider what threshold of risk is acceptable when deciding to pursue adjuvant radiation therapy. The more recent development of clinical tools such as the Memorial Sloan Kettering Cancer Center (MSKCC) Nomogram [11], Oncotype DCIS Score [12], and DCISionRT [13] are used by some physicians to guide patients with this decision. As the treatment approach for good-risk DCIS is very personalized, there exists an exciting opportunity to study real-world patterns of care in this patient population. Furthermore, the development and implementation of partial breast, hypofractionated, and very hypofractionated protocols present even more complexity to the decision-making process.

In this retrospective analysis of the National Cancer Database (NCDB) from 2004 to 2016, we aim to capture a modern-day picture of the patterns of care and use of radiation protocols following lumpectomy in women with "good-risk DCIS."

## Materials and methods

The NCDB - a database supported by the American College of Surgeons and the American Cancer Society-collects de-identified hospital registry data from more than 1,500 Commission on Cancer accredited facilities. Approximately 70% of newly diagnosed cancers in the United States are documented in the NCDB, amounting to 34 million total records. The findings of our study were generated from the NCDB. The sponsors of the NCDB have not verified our study and therefore are not responsible for the conclusions generated. An exemption was obtained from the Research and Development Committee of the Veterans Affairs-New York Harbor Healthcare System prior to the initiation of the study.

First, we isolated 57,790 DCIS patients from the NCDB between 2004 and 2016. Then, we excluded 21,614 patients who either did not undergo surgery or who received a mastectomy. The remaining 36,176 patients were then selected for criteria meeting "good-risk" DCIS (disease measuring <2.5 cm, negative margins, and nuclear grade 1 or 2), establishing a study cohort of 12,846 patients. Among the 12,846 patients, 6,600 received radiation, and 6,246 did not receive radiation.

In order to compare the patients who did and did not receive radiation, the following clinical and patient-related factors were documented: age (<50, 50-65, >65), tumor size (≤1 cm, >1 cm), estrogen receptor (ER) status (ER−, ER+, Unknown), endocrine therapy (Yes, No, Unknown), race (White, Black, and Other), insurance type (None, Private, Medicaid, Medicare, Other/Unknown), and distance from the treatment center stratified into quartiles (0-3.9, 4-8, 8.1-15.8, and >15.8 miles). For the patients who received radiation, the fractionation schedule was collected and categorized as hypofractionation, standard fractionation, or other, if fractionation could not be ascertained.

The Chi-square tests were compared to the frequency distribution of the categorical variables: age, tumor size, ER status, endocrine therapy, margin status, race, insurance type, and distance from the treatment center between the patients who did and did not receive radiation therapy. Chi-square analyses were further used to compare trends in fractionation (hypofractionation versus standard) by year. Multivariate logistic regression analyses were used to identify covariables associated with the use of radiation. All analyses were conducted with SPSS 28.0 (IBM INC., Armonk, New York). All two-sided tests were performed with a p<0.05 significance.

## Results

A total of 12,846 patients met the eligibility criteria. Of those, 6,600 (51.4%) received adjuvant WBRT. 59.8% of patients under the age of 50 received adjuvant RT as compared to 55.8% of patients aged 50-65 and 43.7% of patients older than 65 (P<0.001). Women receiving endocrine therapy were less likely to receive adjuvant RT than those who were not (41.4% vs 61.6%, P<0.001). Patients whose tumors were ER− were significantly more likely to receive adjuvant RT than those whose tumors were ER+ (52.1% vs 42.0%, P<0.001). Insurance status was also significantly correlated with receipt of adjuvant RT (P<0.001). Patients who were uninsured or had private insurance, Medicaid, or "other" insurance were more likely than not to receive adjuvant RT. Patients whose insurance was listed as Medicare were less likely to receive adjuvant RT (44.1% vs 55.9%). Great Circle distance was also correlated with receipt of adjuvant RT (P<0.001). As the distance from the treatment center increased, the percentage of patients receiving adjuvant RT trended down: 53.0%, 52.7%, 51.9%, and 48.0% for each quartile, respectively (Table 1).

**Table 1:** Patient characteristics

Variable	No radiation (N=)	Radiation (N=)	P-value
Age			P<0.001
<50	40.2% (906)	59.8% (1349)	
50–65	44.2% (2276)	55.8% (2876)	
>65	56.3% (3064)	43.7% (2375)	
Endocrine therapy			P<0.001
Yes	58.6% (3472)	41.4% (2451)	
No	38.5% (2412)	61.5% (3852)	
Unknown	54.9% (362)	45.1% (297)	
ER status			P<0.001
ER+	58.0% (690)	42.0% (499)	
ER−	47.9% (5329)	52.1% (5797)	
Unknown	42.7% (227)	57.3% (304)	
Race			P=0.220
White	49.0% (5091)	51.0% (5301)	
Black	47.3% (788)	52.7% (879)	
Other	46.6% (367)	53.4% (420)	
Insurance			P<0.001
Private	44.2% (3099)	55.8% (3905)	
Uninsured	38.1% (59)	61.9% (96)	
Medicaid	41.4% (232)	58.6% (329)	
Medicare	55.9% (2720)	44.1% (2150)	
Other	47.9% (56)	52.1% (61)	
Unknown	57.6% (80)	42.4% (59)	
Distance from treatment center			P<0.001
<4.0 miles	47.0% (1529)	53.0% (1722)	
4.0–8.0 miles	47.3% (1487)	52.7% (1655)	
8.1–15.8 miles	48.1% (1534)	51.9% (1658)	
>15.8 miles	52.0% (1696)	48.0% (1565)	

In multivariable regression, patients over the age of 50 were increasingly less likely to receive adjuvant RT. Specifically, women ages 50-65 had an OR of 0.83 (P<0.001), and those over the age of 65 had an OR of 0.58 (P<0.001). Women not receiving endocrine therapy were significantly more likely to receive adjuvant RT than those who received endocrine therapy (OR 2.24, P<0.001). Women whose tumors were ER− were significantly more likely to receive adjuvant RT than those whose tumors were ER+ (OR 1.24, P<0.001). The race did not have a significant effect on the receipt of adjuvant RT. Among patients with known insurance status, there was no significant effect of insurance on receipt of radiation therapy. Of note, patients with unknown insurance status were significantly less likely to receive adjuvant RT (OR 0.69, P=0.036) as compared to those who had private insurance. Distance >15.8 miles from the treatment center was negatively correlated with receipt of adjuvant RT (OR 0.78, P<0.001) (Table 2).

**Table 2:** Multivariate analysis

Variable	OR	Confidence interval (95%)	P-value
Age			P<0.001
<50	1		
50–65	0.826	0.745–0.916	P<0.001
>65	0.579	0.505–0.663	P<0.001
Endocrine therapy			P<0.001
Yes	1		
No	2.237	2.074–2.413	P<0.001
Unknown	1.132	0.960–1.334	P=0.139
ER status			P<0.001
ER+	1		
ER-	1.236	1.090–1.402	P<0.001
Unknown	2.217	1.794–2.740	P<0.001
Race			P=0.989
White	1		
Black	0.994	0.892–1.107	P=0.909
Other	1.007	0.866–1.170	P=0.932
Insurance			P=0.061
Private	1		
Uninsured	1.294	0.924–1.810	P=0.133
Medicaid	1.098	0.917–1.315	P=0.309
Medicare	0.910	0.809–1.023	P=0.114
Other	0.933	0.640–1.361	P=0.720
Unknown	0.686	0.483–0.975	P=0.036
Distance from the treatment center			P<0.001
<4.0 miles	1		
4.0–8.0 miles	0.980	0.886–1.085	P=0.701
8.1–15.8 miles	0.921	0.833–1.019	P=0.111
>15.8 miles	0.781	0.705–0.863	P<0.001

**Figure 1:**
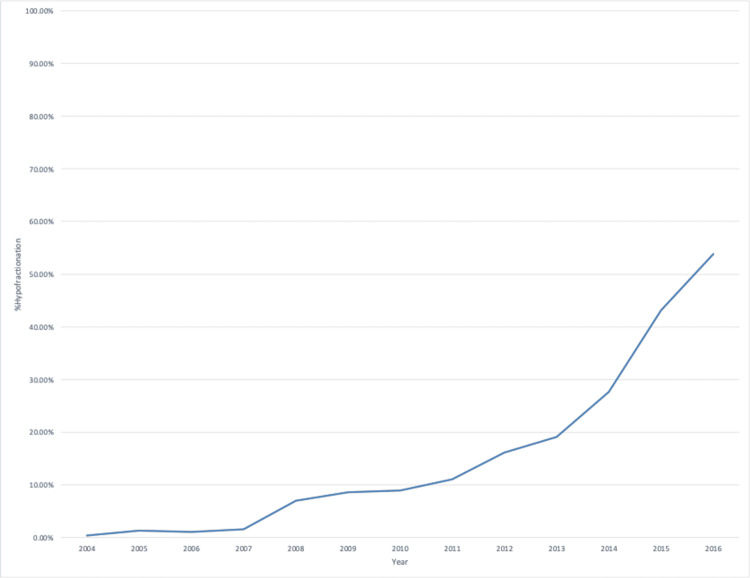
Hypofractionation utilization per year

The fractionation technique was categorized as standard or hypofractionated in 52.2% of patients. For the remaining patients, the total dose or dose per day was not fully recorded or did not compute to a typical radiation dose. Of those, the use of hypofractionation increased from 0.4% in 2004 to 8.9% in 2010 and to 53.8% in 2016 (Figure 1).

## Discussion

DCIS detection has risen with the advent of increasingly sensitive breast cancer screening [14-15]. Unlike invasive cancer, treating DCIS has not been found to correlate with increased overall or breast cancer-specific survival [3-10,16]. Consequently, it is logical for clinicians to attempt to de-escalate care. ECOG E5194 [8] and RTOG 9804 [9] each took related but different approaches. RTOG 9804 selected "good-risk" DCIS patients and randomized them to lumpectomy +/- adjuvant radiation therapy. ECOG 5194 non-randomly assigned patients to favorable or unfavorable cohorts and treated all patients with lumpectomy without adjuvant radiation therapy. At the seven-year follow-up of RTOG 9804, LF was 6.7% and 0.9%, respectively, for the lumpectomy-only arm and the lumpectomy + radiation arm. Recently published 15-year follow-up data showed LF of 15.1% and 7.1%, respectively [10]. ECOG 5194 showed 12-year LF rates of 14.4% and 24.6%, respectively, for the favorable cohort and unfavorable cohort, respectively. Neither of these trials definitively recommended omitting adjuvant radiation therapy but instead suggested that the decision should be an informed discussion with the patient.

In this retrospective NCDB review of "good-risk DCIS" patients treated from 2004 to 2016, 51% of patients received adjuvant radiation therapy. There was no trend regarding receipt of adjuvant radiation therapy with respect to the year. Age was correlated with the receipt of radiation therapy in both univariate and multivariate analyses. As a patient’s age increased, she was less likely to undergo treatment. Studies such as PRIME II and CALGB C9343 have investigated the omission of radiation therapy in older, low-risk, invasive breast cancer patients [17,18]. Much like the aforementioned DCIS trials, the consensus is that treatment decisions should be a discussion with the patient. It is, however, no surprise that if clinicians are omitting adjuvant radiation therapy for low-risk invasive cancer in older patients, these practices may be extrapolated to patients with non-invasive disease.

When deciding whether to omit adjuvant radiation therapy, an important factor is whether the patient plans to undergo additional treatment. The NSABP-24 and UK/ANZ trials each showed decreases in ipsilateral and contralateral breast events in DCIS patients receiving tamoxifen [7,19]. The MSKCC's DCIS nomogram includes both adjuvant radiation therapy and adjuvant endocrine therapy in its risk calculations [11]. NSABP-24 also showed that tamoxifen therapy only benefits patients whose tumors are ER+. Intuitively, in our study, women whose tumors were ER− and those not undergoing endocrine therapy were significantly more likely to receive radiation therapy.

Lastly, the distance to the treatment center significantly affected the receipt of adjuvant radiation therapy. Patients more than 15.8 miles away from the treatment center were significantly less likely in univariate and multivariate analysis to receive adjuvant radiation therapy. Breast radiation therapy typically consists of three to six weeks of daily treatments. While each treatment is brief, the time commitment of round-trip travel as well as time spent in the department may present a barrier for some women. This effect may be especially present in women who are unable to take off work during their treatment. As technology and treatment techniques evolve, newer hypofractionated regimens have emerged that relieve some of this time burden. As of 2018, ASTRO recommends that all patients receiving WBRT without regional nodal irradiation get hypofractionated radiation therapy consisting of 4000-4250 cGy in 15-16 fractions [20]. Our study showed that the employment of hypofractionated regimens has increased rapidly recently, with most patients receiving hypofractionated radiation therapy in 2016. As newer data are released, we expect that this trend will continue.

More recently, even more accelerated radiation therapy regimens have been studied and proven efficacious. Accelerated partial breast irradiation is becoming more common [21-25]. Additionally, the FAST and FAST-Forward trials have looked at five-fraction WBRT in either daily or weekly fractions [26,27]. As these techniques become more common, future reviews could revisit patterns of care as they relate to newer hypofractionated techniques.

This review was limited by the data available from the NCDB. Data are entered by the participating institutions with no central review or validation. It is feasible that some data are entered incorrectly or accidentally omitted or duplicated. The NCDB does not track outcomes other than survival, nor does it provide a complete picture of treatment decision-making. Lastly, the data were only available through 2016, but trends may be changing in more recent years as guidelines now more consistently recommend hypofractionation [2,20].

## Conclusions

In conclusion, from 2004 to 2016, 51.2% of patients who met the criteria for RTOG 9804 "good-risk DCIS" received adjuvant radiation therapy. Receipt of adjuvant radiation therapy was affected by patient age, receptor status, endocrine therapy, and distance from treatment. The utilization of hypofractionated regimens has increased over time. Increased utilization of partial breast radiation or shorter treatment courses may increase the use of radiation for these patients, particularly for those traveling more than 16 miles a day for treatments.
